# Kalopanaxsaponin A induces reactive oxygen species mediated mitochondrial dysfunction and cell membrane destruction in *Candida albicans*

**DOI:** 10.1371/journal.pone.0243066

**Published:** 2020-11-30

**Authors:** Ying Li, Mingzhu Shan, Yao Zhu, Huankai Yao, Hongchun Li, Bing Gu, Zuobin Zhu

**Affiliations:** 1 Xuzhou Key Laboratory of Laboratory Diagnostics, School of Medical Technology, Xuzhou Medical University, Xuzhou, China; 2 Department of Genetics, Xuzhou Medical University, Xuzhou, China; 3 Jiangsu Key Laboratory of New Drug Research and Clinical Pharmacy, School of Clinical Pharmacy, Xuzhou Medical University, Xuzhou, China; 4 Department of Laboratory Medicine, Affiliated Hospital of Xuzhou Medical University, Xuzhou, China; Lebanese American University, LEBANON

## Abstract

Candidiasis causes high morbidity and mortality among immunocompromised patients. Antifungal drug resistance and cytotoxicity highlight the need of effective antifungal therapeutics. In this study, we found that kalopanaxsaponin A (KPA), a triterpenoid saponin natural product, could inhibit the proliferation of various *Candida* species, and exerted a fungicidal effect against *C*. *albicans*. To further explore its antifungal action mode, spectrofluorophotometer, fluorescence microscopy and transmission electron microscopy were performed, showing that KPA treatment induced the accumulation of intracellular reactive oxygen species (ROS), resulting in mitochondrial dysfunction. Meanwhile, KPA treatment also broke down the membrane barrier of *C*. *albicans* causing the leakage of intracellular trehalose, the entrance of extracellular impermeable substance and the decrease of ergosterol content. Both ROS accumulation and membrane destruction contributed to the death of *C*. *albicans* cells. Our work preliminarily elucidated the potential mechanisms of KPA against *C*. *albicans* on a cellular level, and might provide a potential option for the treatment of clinical candidiasis.

## 1. Introduction

*Candida albicans* is the major pathogenic source of candidiasis worldwide accompanying with other non-*albicans Candida* species, including *C*. *glabrata*, *C*. *parapsilosis*, *C*. *tropicalis* and *C*. *krusei* [1,2]. Candidiasis occurs in the setting of some infectious risk factors at any age, even superficial infection, deep-seated infection and bloodstream infection in immunocompromised or immunologically deficient individuals. Invasive candidiasis is closely linked to the advanced medical technology and has high morbidity and mortality (~ 40%) in nosocomial infections [3]. Even with the treatment of the antifungal agents available, by far, candidiasis remains the most common fungal infection disease, ranking the third-to-fourth most frequent nosocomial infections in the United States [4]. Furthermore, long-term antifungal therapy and biofilms formed on both biological and inert surfaces accelerate the development of drug resistance, complicating the management of candidiasis [5,6]. Additionally, resistance and cytotoxicity are the inevitable problems for most antifungal drugs [7,8]. Hence, novel antifungal drugs or therapeutic strategies are urgently need for the clinical treatment of candidiasis.

**Fig 1:**
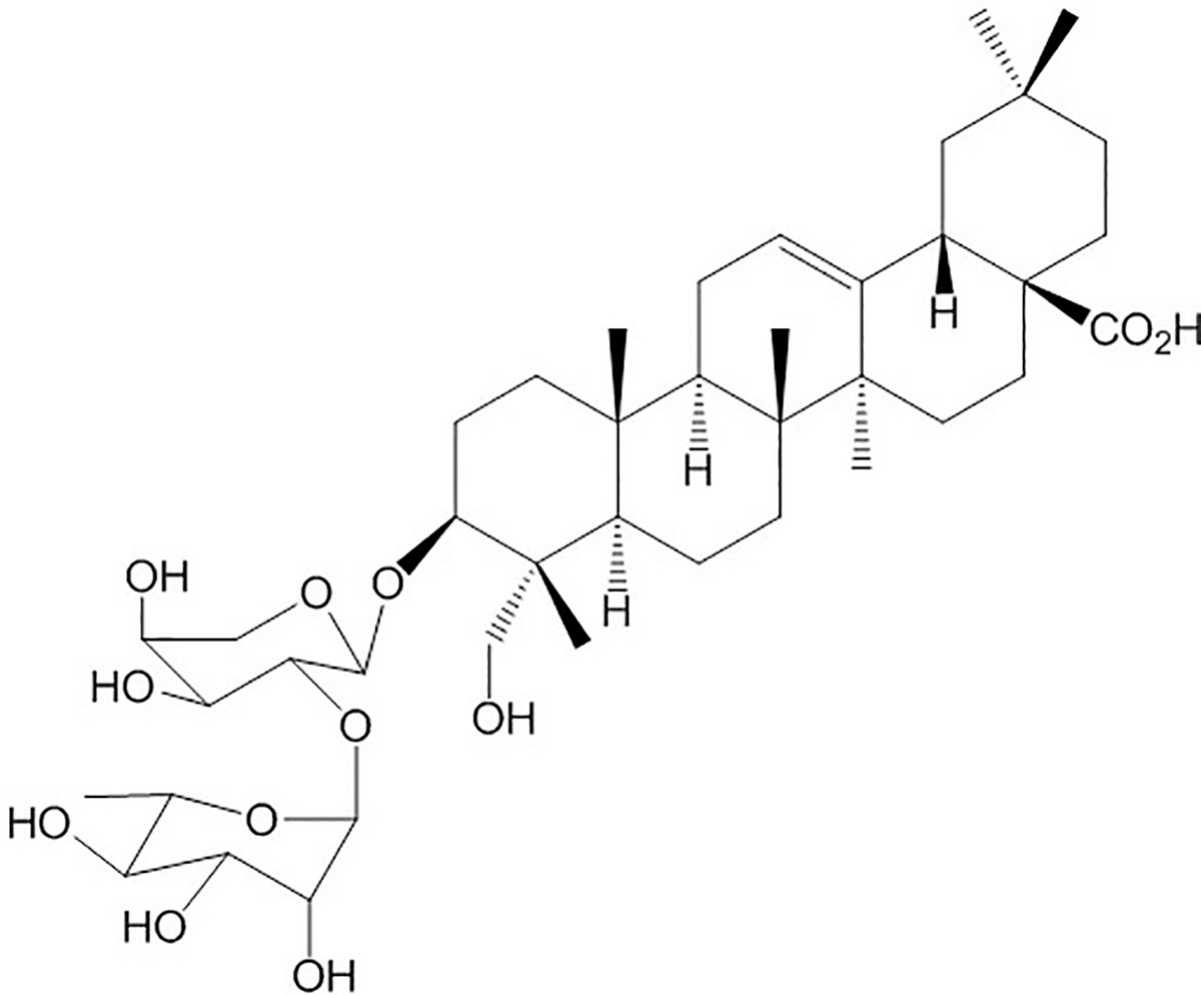
The structure of KPA.

Natural products have long been regarded as ample sources for novel drugs with pharmacological properties, and over half of the new drugs approved by the U.S. Food and Drug Administration (FDA) are either natural products or based thereon [9]. As our lab reported previously [10], kalopanaxsaponin A (KPA, **Fig 1**), a triterpenoid saponin from the stem bark of *Kalopanax pictus*, could effectively retard the virulence of *C*. *albicans* with low cytotoxicity, especially in the process of morphological switch. In this study, we found that KPA could inhibit the proliferation of various *Candida* species and exert fungicidal activity against *C*. *albicans*, the most common clinical fungal pathogen. Redox-related physiological alterations are known as part of microbial lethality caused by antibiotics [11]. Our further research found that both the accumulation of intracellular reactive oxygen species (ROS) and damage to the cell membrane contributed to the fungicidal activity of KPA against *C*. *albicans*. The present study is intended to clarify its mode of action in *C*. *albicans* after KPA treatment, and provide an alternative antifungal natural product for the discovery of new antifungal drugs.

## 2. Materials and methods

### 2.1 Strains, culture and chemicals

Five most common *Candida* species were used in this study, including *C*. *albicans* (SC5314 and CA171113-9), *C*. *glabrata* (CG171122302), *C*. *parapsilosis* (CP18092240), *C*. *tropicalis* (CT171114-7) and *C*. *krusei* (CK1) **(Table 1**). Clinical isolated strains were kindly provided by the affiliated hospital of Xuzhou Medical University (Xuzhou, China). The strains were maintained at -80°C. Before each experiment, cells were routinely prepared as previously described [10].

**Table 1:** The MICs values of KPA against different *Candida* species.

Strains^α^	MICs of drugs (μg/mL)	
	KPA	AMB
SC5314	16	0.5
CA171113-9	16	0.5
CG171122302	16	1
CP18092240	16	0.5
CT171114-7	16	0.5
CK1	16	0.5

^α^SC5314 is wild-type *C*. *albicans* strain. CA171113-9 is a clinical isolated azoles multi-resistance *C*. *albicans* isolate. CG171122302 is a clinical isolated *C*. *glabrata* isolate. CP18092240 is a clinical isolated *C*. *parapsilosis* isolate. CT171114-7 is a clinical isolated *C*. *tropicalis* isolate. CK1 is a clinical isolated *C*. *krusei* isolate.

KPA was separated from the stem of the *Kalopanax* in our laboratory with a purity of over 98% as analyzed by the Agilent Infinity 1220 LC system (Agilent Technologies, USA). Amphotericin B (AMB), dimethyl sulfoxide (DMSO), 2’,7’-Dichlorofluorescin diacetate (DCFH-DA), rhodamine 123 (Rh123), thiourea (Tu), Vitamin C (VC) and Vitamin E (VE), propidium iodide (PI) were all purchased from Sigma (St Louis, MO, USA). KPA, AMB and DCFH-DA were dissolved in DMSO, and Rh123, Tu, VC, VE and PI were dissolved in sterile water at a concentration of 10 mg/mL and frozen at -20°C until use. In each assay, the content of DMSO was below 1%.

### 2.2 Antifungal susceptibility test

The 80% Minimum Inhibitory Concentration (MIC_80_) values of KPA against various *Candida* species were detected by the broth microdilution method according to the Clinical and Laboratory Standards Institute (CLSI) guidelines (M27-A3) [12]. AMB served as positive control. The minimal concentration of zero visible growth was considered as the endpoint value.

### 2.3 Time-killing kinetics test

To evaluate the fungicidal action of KPA against *C*. *albicans* SC5314 over time, the time-killing kinetics curves were plotted by measuring the cell survival rates under KPA (16 μg/mL) or AMB (1 μg/mL, positive control) treatment as previously described with some modification [13]. SC5314 cells (1 × 10^6^ cells/mL), suspended in synthetic medium plus dextrose (SD medium), were respectively incubated with KPA or AMB at 30°C, 200 rpm. After 12 h incubation, the treated cells were put back to fresh SD medium and cultured until 48 h under the same condition. The number of viable cells was determined by the colony counting method every two hours.

### 2.4 Measurement of intracellular ROS generation

DCFH-DA was used to detect the level of intracellular ROS as previously reported [13,14]. SC5314 cells were treated with series concentrations of KPA or 1 μg/mL of AMB (positive control) in SD medium. After 3 h incubation at 30°C, cells were harvested and washed three times. Then, cells were co-incubated with 40 μg/mL of DCFH-DA for 30 min in the dark. After three times washes with sterile PBS, the fluorescence intensity of cells were measured with a BioTek Synergy2 spectrofluorophotometer (BioTek Instruments, USA) at 486 nm excitation and 525 nm emission wavelengths and observed using an Olympus BX53F fluorescence microscope (Olympus, Tokoyo, Japan). The effect of antioxidant Tu, a ROS scavenger, on the ROS generation with or without KPA treatment was also tested by pre-treatment with 5 mM Tu at 30°C for 1 h according to the previously described method [13].

### 2.5 Detection of mitochondrial function

Knowing that mitochondrial membrane potential (mtΔ*ψ*) and intracellular ATP levels are important indicators of the mitochondrial structure and function, we examined the effect of KPA on the alteration of mtΔ*ψ* and ATP content as previously reported with some modification [13,14]. SC5314 cells (1 × 10^6^ cells/mL) were treated with different concentrations of KPA in SD medium at 30°C for 3 h and then stained with 5 μM Rh123 for 30 min in the dark, after three times washes with sterile PBS, cells were measured with a BioTek Synergy2 spectrofluorophotometer at 486 nm excitation and 525 nm emission and observed with the Olympus BX53F fluorescence microscope. This experiment was also carried out in the presence of 5 mM Tu as previously reported [13].

Intracellular ATP levels were measured according to the previously reported method [14]. Treated SC5314 cells were disrupted by vortexing with glass beads, and the intracellular ATP levels were determined using an ATP Assay kit (Beyotime, Haimen, Jiangsu, PR China) according to the provided protocol.

### 2.6 Effect of the antioxidant on fungicidal activity of KPA

SC5314 cells (1 × 10^6^ cells/mL) were pre-incubation with or without Tu (5 mM), VC (10 mM) or VE (10 mM) in SD medium respectively for 1 h at 30°C according to previously described method [13,14]. KPA was then added with a final concentration of 16 μg/mL before the incubation at 30°C for additional 3 h. Drug free treatments with or without Tu, VC or VE served as control groups. Cells were taken at 1, 2 or 3 h, and the number of viable cells was determined by spot assay.

### 2.7 Determination of intracellular and extracellular trehalose content

SC5314 cells (1 × 10^6^ cells/mL in SD medium) were treated with different concentrations of KPA (0, 8, 16 and 32 μg/mL) at 30°C for 3 h to determine the change of intracellular and extracellular trehalose content according to previously described with some modification [15]. 1 μg/mL of FLC served as the positive control. Subsequently, cells were separated by centrifugation. The pellets were dried and weighted to test the intracellular trehalose level, and the supernatants were transferred to a new tube to test the extracellular level. After disrupting cells by vortexing with glass beads, the intracellular and extracellular trehalose level was determined with a trehalose assay kit (Beijing Solarbio Science & Technology Co., Ltd., China) according to the provided protocol.

### 2.8 PI staining

PI, a membrane impermeable fluorescent dye, could only penetrate destroyed cell membrane and stain intracellular DNA with red fluorescence. We used PI to detect the damage of membrane induced by KPA [13,15]. Treated SC5314 cells were washed three times and re-suspended with PBS. After stained with 5 μM PI in the dark for 10 min and three washes, the images of cells were taken by an Olympus BX53F fluorescence microscope to calculate the ratio of stained cells.

### 2.9 Determination of ergosterol content

SC5314 cells (1 × 10^6^ cells/mL in SD medium) were treated with different concentrations of KPA (0, 8, 16 and 32 μg/mL) at 30°C for 3 h to determine the ergosterol content of cell membrane using the alcoholic KOH method according to previously described [16]. 1 μg/mL of FLC served as the positive control. The extract was scanned spectrophotometrically between 240 and 300 nm (Shimadzu UV-2450, Japan). The ergosterol content was calculated with the following equations: % ergosterol = [(A_281.5_/290) × F]/pellet weight–[(A_230_/518) × F]/pellet weight, where F is the factor for dilution.

### 2.10 Transmission electron microscopy (TEM)

SC5314 cells were treated with or without 16 μg/mL KPA in SD medium at 30°C for 3 h. Then, cells were centrifuged, washed, fixed, desiccated and embedded as previously described [13,17]. The ultrastructure images of cells were digitally recorded with a transmission electron microscope (Tecnai G2 Spirit TWIN, FEI, Holland).

### 2.11 Statistical analysis

All experiments were assayed in triplicate on a given day and repeated on additional two days. Results are represented as the mean values with standard deviations of triplicate measurements from three independent experiments. The Student *t*-test (two-tailed) was used to analyze the significance of differences between two experimental groups. The log-rank (Mantel-Cox) test was used to analyze the significance differences of time-killing curves between KPA and AMB. Data were analyzed by Origin 9.0 software, and *P* value < 0.05 was considered to be statistically significant.

## 3. Results

### 3.1 Antifungal activity of KPA on *Candida* species (MICs)

The MIC_80_ values of KPA against different *Candida* species were measured by the broth microdilution method. The results showed that KPA exerted a moderate antifungal effect (based on the previous reports [6,10,13,17]) against the five most common *Candida* species, including *C*. *albicans*, *C*. *glabrata*, *C*. *parapsilosis*, *C*. *tropicalis* and *C*. *krusei*, the MIC_80_ value of which was all 16 μg/mL. The MIC_80_ values of the positive control AMB were 0.5 or 1 μg/mL, respectively (**Table 1**).

### 3.2 Effect of KPA on the growth of *C*. *albicans*

**Fig 2:**
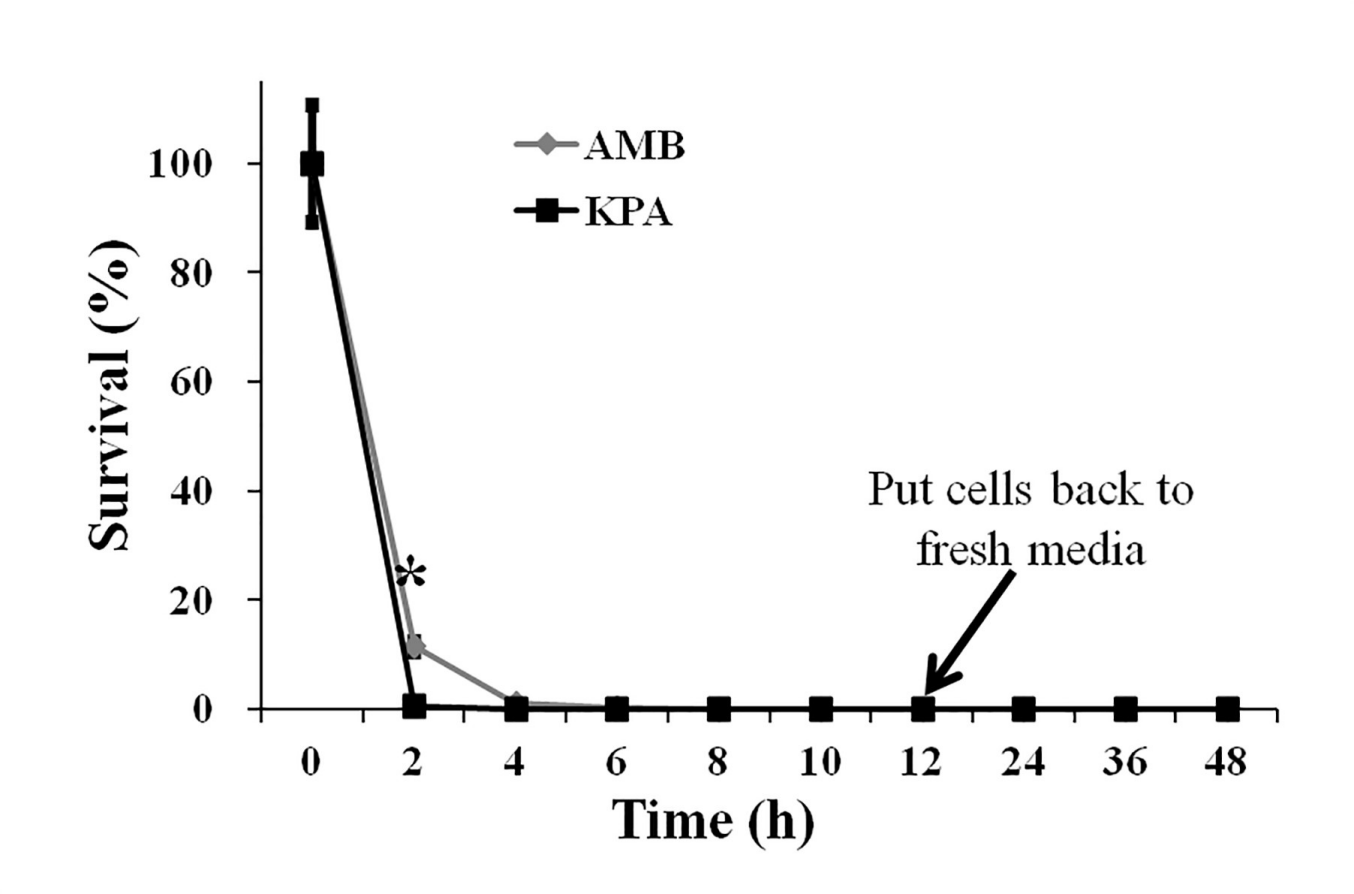
The time-killing curves of KPA and AMB against wild type *C*. *albicans* strain SC5314. Data represent mean values and error bars show standard deviations.

Subsequently, the time-killing curves of KPA (16 μg/mL) and AMB (1 μg/mL) against *C*. *albicans* were depicted based on the colony counting data. As shown in **Fig 2**, there was no significant difference between KPA and AMB treatments (*P* > 0.05), both KPA and AMB could effectively kill wild type *C*. *albicans* SC5314 cells within two hours. After 12 h treatment, put cells back to fresh media, both KPA and AMB treated cells did not revive until 48 h. These results implied that KPA exerted a fungicidal effect against *C*. *albicans* and could inhibit its proliferation in two hours.

### 3.3 Effect of KPA on intracellular ROS generation of *C*. *albicans*

**Fig 3:**
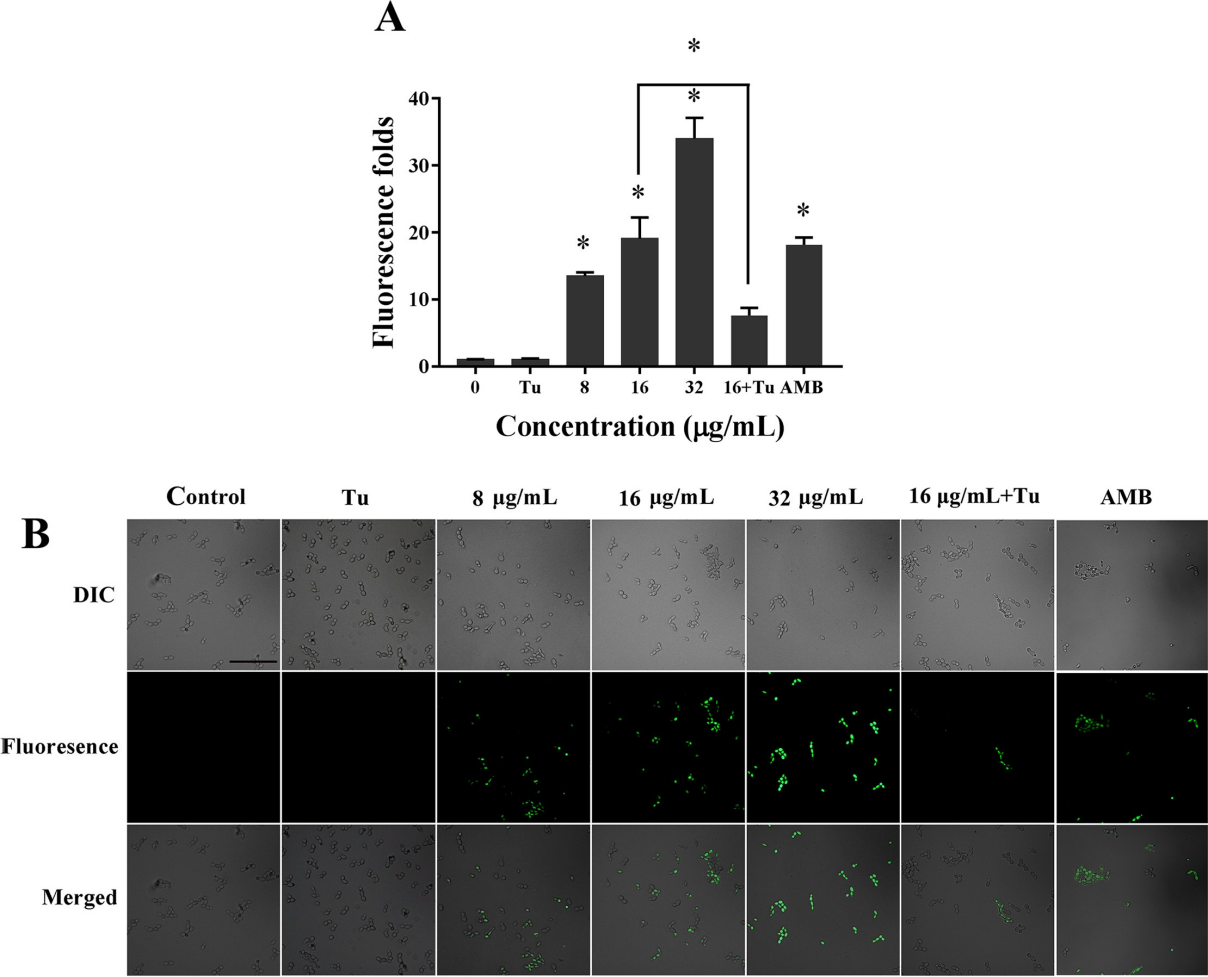
Effect of KPA on intracellular ROS accumulation. **(A)** The relative fluorescence intensity of treated cells stained by DCFH-DA was measured by a BioTek Synergy2 spectrofluorophotometer. Data represent mean values and error bars show standard deviations. * means statistically significant difference (*P* < 0.05). **(B)** The fluorescence microscopic observation of *C*. *albicans* SC5314 with different treatments stained with DCFH-DA. Bar, 50μm.

Fluorescence probe DCFH-DA was used to detect the level of intracellular ROS as it can be oxidized to the highly green fluorescent DCF by ROS [18,19]. As shown in **Fig 3A**, the green fluorescence degree of cells were enhanced markedly with the increase of KPA doses, implying that intracellular ROS was increasingly accumulated. Compared with the drug-free group, 8, 16 and 32 μg/mL KPA significantly increased the ROS generation by 13.48, 19.08 and 34.00 fold, respectively. The 1 μg/mL positive control AMB increased by 18.14 fold. The similar tendency was also observed in the microscopic observation images. KPA treatment resulted in more cells stained in green fluorescence, implying the increase of ROS generation (**Fig 3B**). These results demonstrated that KPA could induce intracellular ROS accumulation.

### 3.4 Effect of KPA on mitochondrial function

Knowing that mitochondria have long been established as a major source of ROS in aerobic cells, we following detected the effect of KPA on the mitochondrial function via detecting the alteration of mtΔ*ψ* and the intracellular ATP concentration.

**Fig 4:**
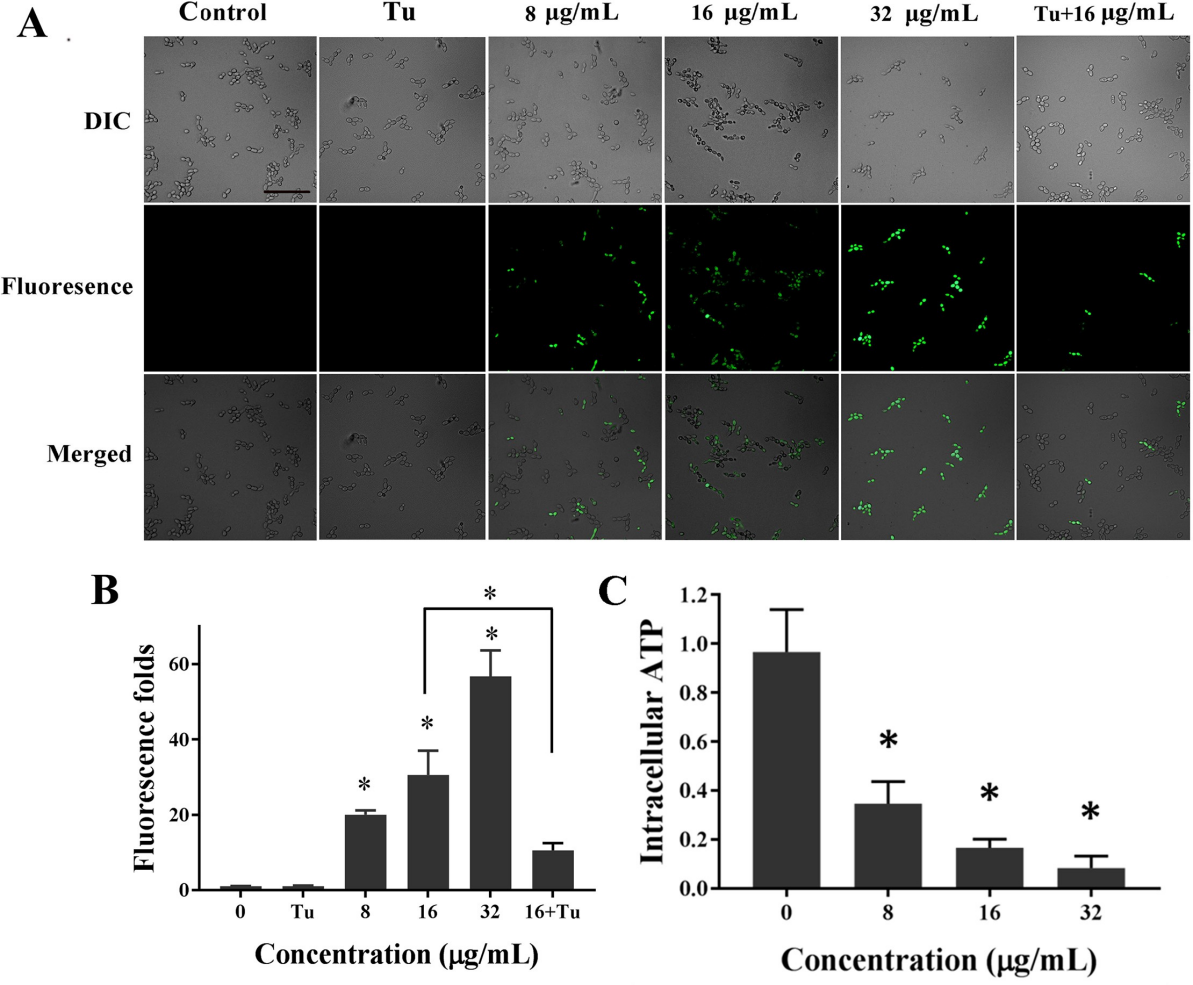
Effect of KPA on the mitochondrial function. **(A)** The fluorescence microscopic observation of *C*. *albicans* SC5314 with different treatments stained with Rh123. Bar, 50μm. **(B)** Change in relative fluorescence intensity of treated SC5314 cells was monitored by spectrofluorophotometry. **(C)** Intracellular ATP was extracted from cells treated with KPA or not and determined using an ATP assay kit. Each column in **(B)** and **(C)** represents the mean values of each group, and error bars show standard deviations. * means statistically significant difference (*P* < 0.05).

We used Rh123, a potential-dependent distributional probe, to determine the mtΔ*ψ* of KPA treated cells [13]. The fluorescence microscopic pictures displayed that the number of cells with green fluorescence was increased in a dose-dependent manner (**Fig 4A**). The spectrofluorophotometer results showed that the fluorescence intensity of KPA-treated cells was also positively correlated with the increased doses (**Fig 4B**). Compared with the drug-free group, 8, 16 and 32 μg/mL KPA significantly increased fluorescence level by 19.96, 30.56 and 56.72 fold, respectively.

An ATP assay kit was used to measure the level of intracellular ATP content. As shown in **Fig 4C**, KPA treatment caused a significant decrease in the intracellular ATP level. In detail, 8, 16 and 32 μg/mL KPA decreased the level of ATP by 0.35, 0.17 and 0.08 fold, respectively.

The above results demonstrated that the mitochondrial function was impaired by the KPA treatment.

### 3.5 Effect of antioxidant on the antifungal activity of KPA

**Fig 5:**
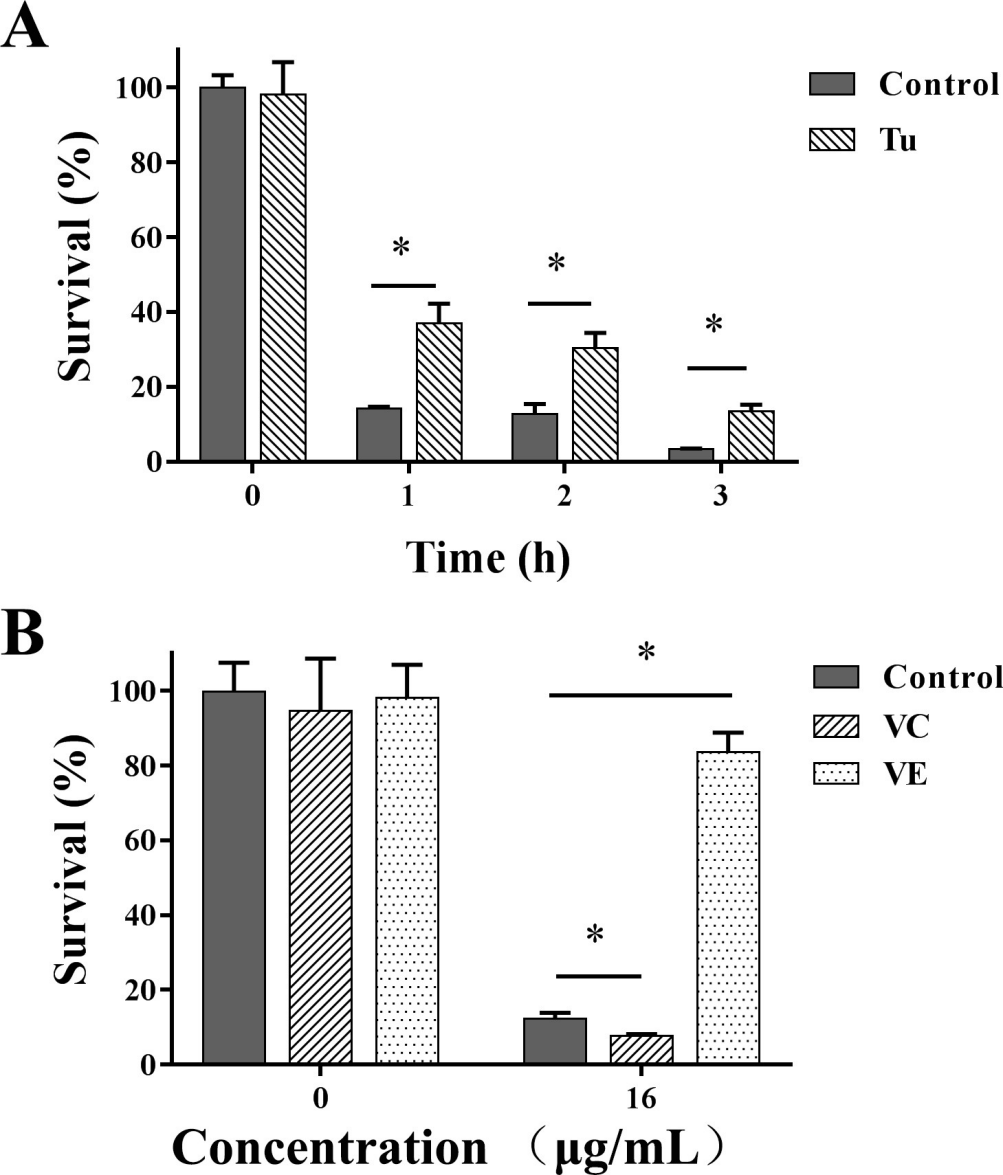
Effect of different antioxidant Tu, VC and VE on the fungicidal activity of KPA. SC5314, pretreated with or without Tu **(A)**, VC **(B)** or VE **(B)** was exposed to 16 μg/mL of KPA for 3 h at 30°C. The number of viable cells was then determined by colony counting method. The bars indicate standard deviations. * means statistically significant difference (*P* < 0.05).

Tu, a ROS scavenger, was utilized to determine whether ROS accumulation was directly responsible for the fungicidal activity of KPA. As shown in **Figs 3** and **4**, Tu pretreatment had little effect on the intracellular ROS generation and the mtΔ*ψ* level in normal *C*. *albicans* cells. However, Tu could significantly retard KPA-induced ROS accumulation and mtΔ*ψ* hyperpolarization. In addition, the fungicidal activity of KPA was partially hindered by the addition of Tu. As shown in **Fig 5A**, the antioxidant Tu had little effect on the growth of normal cells, on the contrary, Tu significantly increased the survival percentage of KPA-treated cells by more than 2 fold after 1, 2 or 3 h incubation as compared with KPA (16 μg/mL) action alone.

VC and VE are two popular antioxidant nutrients that easily exist in human blood or skin without significant toxicity through oral administration or makeup [20]. Therefore, the effect of VC or VE on the antifungal activity of KPA was also tested by the colony counting method. The results showed that VC or VE pretreatment did not affect normal cells proliferation. However, the supplement of VC effectively enhanced the fungicidal efficacy of KPA as represented by the survival rate of KPA treated cells decreasing from 12.33% to 7.76%, whereas the combination of KPA with VE increased the survival rate to 83.67% (**Fig 5B**).

The above results indicated that KPA exerted its fungicidal activity, at least partially, by stimulating intracellular ROS generation to cause mitochondrial dysfunction. VC and VE had opposite effects on the fungicidal activity of KPA.

### 3.6 Effect of KPA on the cell membrane of *C*. *albicans*

Ample evidence shows that the mitochondrial dysfunction induced by elevated ROS in *C*. *albicans* is associated with the damage of cell membrane, knowing that cell membrane is susceptible to the toxic effects of chemicals [13,21]. Therefore, the effect of KPA on the cell membrane of *C*. *albicans* was explored further by detecting the intracellular and extracellular trehalose content, the permeability of fluorescent dye PI and the content of ergosterol in cell membrane.

**Fig 6:**
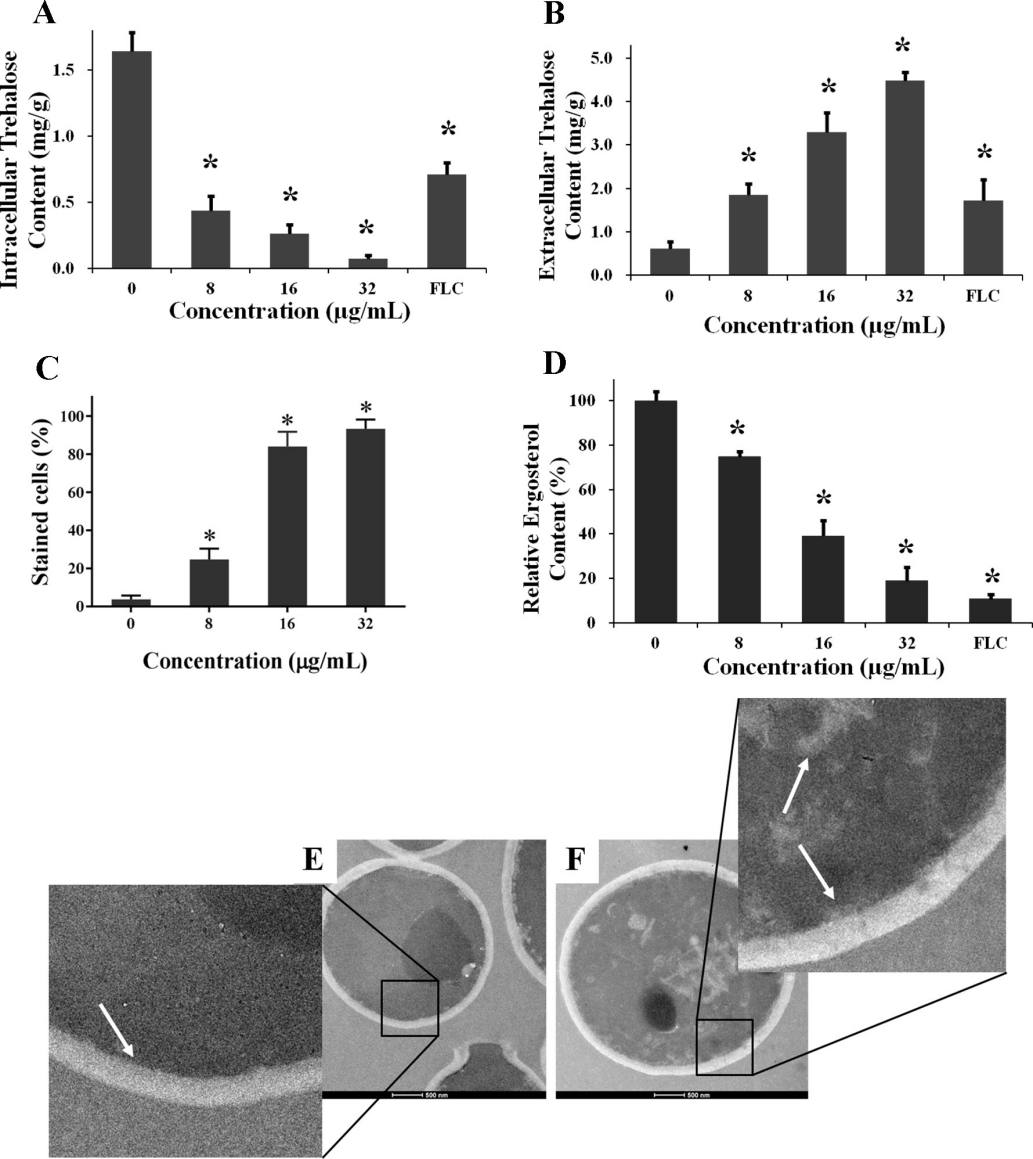
Effect of KPA on the cell membrane of *C*. *albicans*. **(A)** Effect of KPA on the intracellular trehalose content. **(B)** Effect of KPA on the extracellular trehalose content. **(C)** Effect of KPA on the change of membrane permeabilization by PI staining. **(D)** Effect of KPA on the ergosterol content. Data in **(A)**, **(B), (C)** and **(D)** represent mean values, and bars are standard deviations. * means statistically significant difference (*P* < 0.05). The ultrastructure of KPA-treated *C*. *albicans* cells was shown in **(E)** and **(F)**. As indicated by the white arrow in **(E)** and **(F)**, comparing with control cells in **(E)**, cell membrane destruction and disintegration were observed in KPA-treated cells. Bars, 500nm.

We measured the intracellular and extracellular trehalose content under different concentrations of KPA treatment. Results showed a significant decrease in the content of intracellular trehalose (**Fig 6A**) and a significant increase in the content of extracellular trehalose (**Fig 6B**). In detail, the content intracellular trehalose in drug free group was 1.64 mg/g. Whereas, 8, 16 and 32 μg/mL KPA groups were 0.44, 0.26 and 0.07 mg/g, respectively, and the positive control FLC group was 0.71 mg/g (**Fig 6A**). The extracellular trehalose content in drug free group was 0.61 mg/g. 8, 16 and 32 μg/mL KPA groups were 1.86, 3.29 and 4.49 mg/g, respectively, and the FLC group was 1.72 mg/g (**Fig 6B**). These results implied the leakage of intracellular trehalose induced by the destruction of membrane permeability.

PI, a cell membrane impermeable fluorescent dye, was used to verify the damage of membrane permeabilization. The results showed that KPA treatment increased the percentage of PI stained cells in a dose-dependent manner. In detail, the percentage of stained cells in the control group was significantly lower than that in 8, 16, and 32 μg/mL groups (3.67% vs. 24.67%, 84.00% and 93.33%, *P*< 0.05) (**Fig 6C**).

Ergosterol is one of the most important sterol present in cell membrane and plays an important role in growth and development of *Candida* species [22]. As shown in **Fig 6D**, KPA induced significant decrease in ergosterol content of cell membrane. Comparing with drug free group, 8, 16 and 32 μg/mL KPA treatments reduced the ergosterol contents by 74.71%, 39.13% and 18.85%, respectively, and the positive control FLC group was 10.59%.

The above results showed that KPA could damage the permeability of cell membrane and break down the barrier of membrane.

### 3.7 Effect of KPA on the ultrastructure of *C*. *albicans*

The ultrastructure of the *C*. *albicans* cells in response to 16 μg/mL KPA treatment was observed by TEM. Ultrastructure pictures displayed that untreated cells had a normal cellular morphology with distinct cell membranes and intact membranous organelles. The cell membrane was smooth (white arrow, **Fig 6E**). In contrast, the plasma membrane was completely destroyed in KPA treated cells. No distinct cell organelles were observed, and some membrane fragments appeared in the treated cell (white arrows, **Fig 6F**).

## 4. Discussion

KPA is an interesting natural product that exhibited various pharmacological activities in preclinical experimental models [10,23–25]. We previously reported that KPA could retard various virulence factors of *C*. *albicans in vitro*, and high doses of KPA did not affect the survival of healthy *Caenorhabditis elegans*. In addition, it could significantly extend the life time of *C*. *albicans*-infected *C*. *elegans*. Further research found that KPA could promote the secretion of farnesol and decrease the intracellular cAMP level, which together inhibited the morphological transition and biofilms formation of *C*. *albicans* [10]. In this study, we found that KPA presented antifungal activity against five most common *Candida* species and exerted fungicidal activity against *C*. *albicans* in two hours, which is as quick as clinical antifungal drug AMB. More importantly, KPA treated cells could not revive after put back to fresh media within 48 h. All the above results suggest that KPA might be a potential clinical therapeutic option for candidiasis. To better understand how KPA works on a cellular level, we subsequently explored its molecular antifungal mechanisms.

It is generally known that the production of ROS is a universal action mechanism of antifungal drugs against pathogenic fungi, such as AMB, contributing to their fungicidal activities [26]. Farnesol, a quorum sensing molecule in *C*. *albicans*, could induce the generation of ROS by inhibiting the mitochondrial electron transport chain, while KPA could induce the secretion of farnesol in *C*. *albicans* as we have previously reported [27]. Therefore, we firstly detected the effect of KPA on the intracellular ROS level, and found that KPA treatment induced ROS accumulation in a dose-dependent manner.

As cellular metabolism depends on the continuous supply of ATP from the mitochondria, any damage impairing the function of respiratory chain might also impact cell viability. Mitochondria are intimately linked to their role as the major intracellular source of ROS, which are mainly generated at mitochondrial respiratory chain. At the same time, mitochondria are an important target for the damaging effects of excessive ROS production via the disruption of electron transport, mtΔ*ψ* and ATP generation [28,29]. In this study, the hyperpolarization of mtΔ*ψ* and the decrease of ATP production indicated that KPA induced ROS accumulation could cause mitochondrial dysfunction.

**Fig 7:**
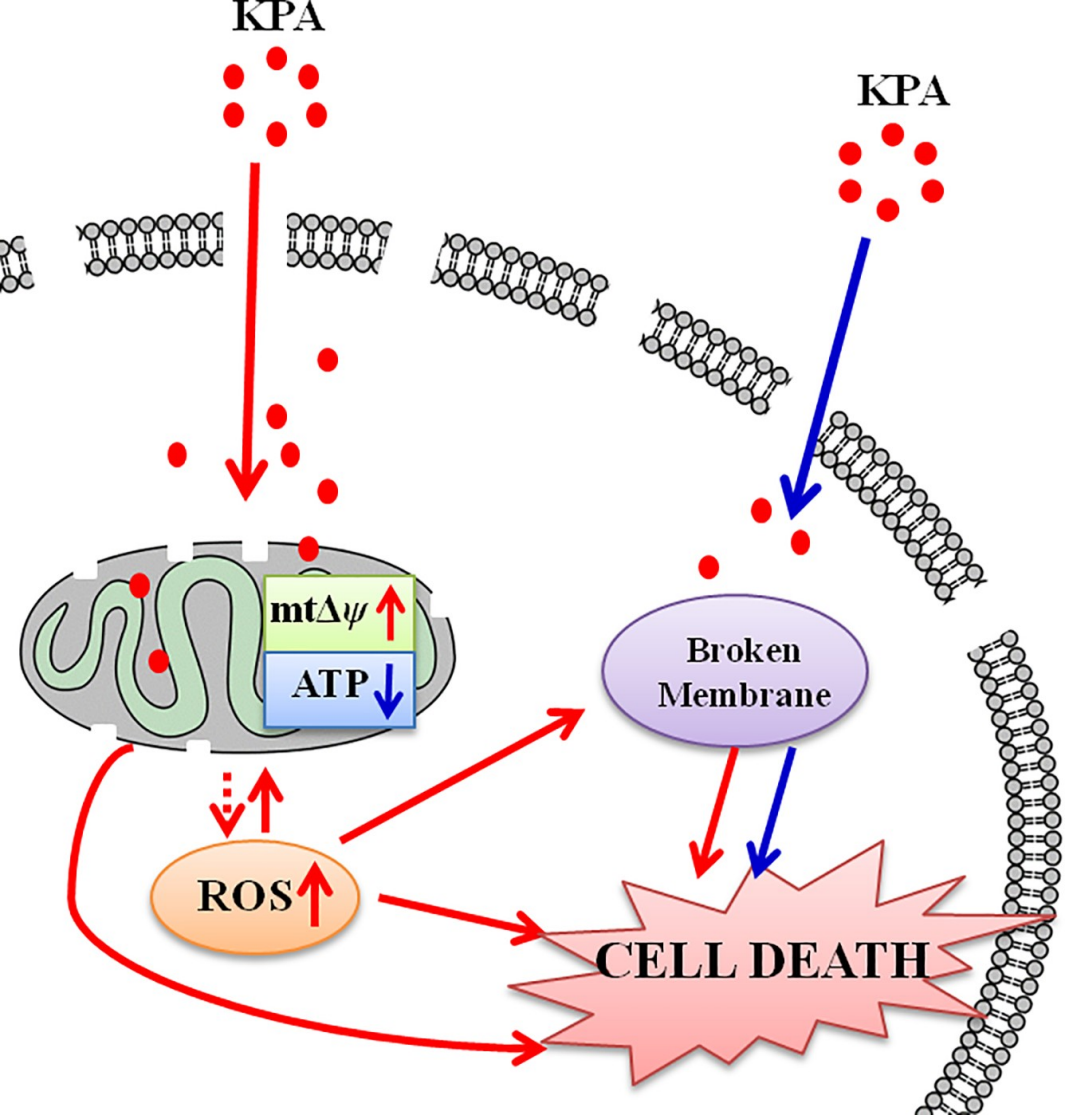
The action model of KPA in *C*. *albicans*. KPA stimulated mitochondria to generate more ROS. Excessive ROS in turn damage the function of mitochondria. The cellular membrane permeability barrier can be broken by the elevated ROS or KPA directly. Mitochondria dysfunction, ROS accumulation and membrane destruction all contribute to *C*. *albicans* cell death.

To confirm the role of ROS on the fungicidal activity of KPA, Tu, a well-known antioxidant, was used. We found that the addition of Tu could lessen KPA-induced ROS accumulation and mtΔ*ψ* hyperpolarization. More importantly, Tu could effectively increase the survival rates of KPA treated cells as compared with the control group, suggesting that the retardation of ROS accumulation could relieve the oxidative stress on mitochondria, and then partly weaken the fungicidal activity of KPA. Taken together, it is concluded that KPA initially stimulates mitochondria to produce extra ROS for anti-stress until overwhelming the limit of cellular antioxidant capacity. Next, excessive ROS in return damages the function and respiratory chain of mitochondria in return, which ultimately contributes to the death of *C*. *albicans* (**Fig 7**).

VC and VE, two known natural antioxidants, can terminate free radicals by eliminating the unpaired condition of the radical [30]. We observed an interesting phenomenon that VC and VE had opposite effects on the fungicidal activity of KPA. More detailedly, VC potentiated the antifungal activity of KPA, while VE reduced its effectiveness against *C*. *albicans*. As reported in other antifungal natural products, such as honokiol, the molecular mechanisms of these opposite effects have been reported by Sun Lingmei et al. [14]. This discovery reminds the synergistic or antagonistic effect between KPA and VC or VE again, which may guide the clinical application of KPA to avoid VE and combine VC.

Cell membrane sets a permeability barrier to protect cells against external stress, which is crucial for cell survival. As reported, intracellular free radicals could induce oxygen toxicity to damage the cell membrane, and mitochondria-perturbing agents could decrease sterol content of the membrane by perturbing respiratory chain to reduce intracellular ATP concentrations. Therefore, excessive ROS always not only leads to the oxidation of macromolecules but is accompanied with the damage to the cell membrane [31–33]. We also observed the KPA-induced damage of cell membrane in this study.

Trehalose exists in eukaryotic microorganisms, in which it may serve as a signaling molecule to regulate certain metabolic pathways, and protect cellular protein or membrane against inactivation by some stress conditions, such as desiccation, dehydration, heat, cold, oxidation and toxic agents [34]. PI, a cell membrane impermeable fluorescent dye, cannot stain normal cells because of the cell membrane barrier. However, it can enter the destroyed membrane of necrotic cells to stain cells in red fluorescence by combining intracellular DNA [35]. Ergosterol, a unique component in the cell membrane of fungus, involved in various physiological functions of *Candida* species, such as drug resistance, morphogenesis and so on [22]. It is crucial for the survival of fungus.

In this study, the decreased intracellular trehalose and ergosterol content and the increased extracellular trehalose content and PI stained cells suggest that the membrane permeability barrier is broken down during KPA exposure. It's worth noting that the total content of trehalose (intracellular plus extracellular) were increased under KPA treatment, suggesting the KPA induced cell stress for *C*. *albicans*. The destruction of cell membrane and organelles membrane was also demonstrated by the TEM images of KPA treated cells. It is possible that the structure of membrane is directly perturbed by KPA treatment to form pores in the membrane as well as causing the leakage of intracellular components and the entrance of extracellular substance to accelerate the death of *C*. *albicans*. Furthermore, damage to the membrane might also be induced by excessive ROS (**Fig 7**).

Above all, we preliminary elucidated the potential mechanisms of KPA against *C*. *albicans*. KPA may stimulate the generation of ROS directly or indirectly to result in ROS accumulation, and then the excessive ROS induces mitochondria dysfunction by perturbing their respiratory chains, which finally damages the permeability of the cell membrane barrier, whereas it is also possible that KPA directly destroys the cell membrane devoid of the ROS pathway. Both ROS accumulation and cell membrane destruction are two important reasons for the fungicidal activity of KPA. Our work uncovered the antifungal molecular mechanisms of KPA *in vitro*, nevertheless, *in vivo* studies in mice need to be carried out to determine its biocompatibility, cytotoxicity, safety and mode of action before further biomedical applications.
